# Congenital Pulmonary Stenosis

**Published:** 1902-08

**Authors:** Joseph Burke

**Affiliations:** Visiting Physician to the Emergency Hospital of the Sisters of Charity; attending physician to the St. Frances’ Asylum for the Aged; attending physician to the St. Vincent’s Female Orphan Asylum; consulting physician to the Widows’ and Orphans’ Asylum of the Sisters of Charity, etc., of Buffalo, N. Y.


### Congenital Pulmonary Stenosis.[1]

By JOSEPH BURKE, Sc. M., M. D.,

Visiting Physician to the Emergency Hospital of the Sisters of Charity; attending physician to the St. Frances' Asylum for the Aged; attending physician to the St. Vincent's Female Orphan Asylum; consulting physician to the Widows' and Orphans' Asylum of the Sisters of Charity, etc., of Buffalo, N. Y.

UNTIL about the middle of the 18th Century the observations upon the congenital heart lesions are found classified under the title of pathological-anatomical rarities and they excited as such, therefore, on account of the lack of knowledge of the symptoms, intra vitam, no clinical interest. Since this time, however, the communications, as from Morgagni, Sandi-

1. Read before the Medical Society of the County of Erie, June 17, 1902.

fort, Hunter, Peacock and others concerning cases of this nature, particularly concerning the examinations of malformations, which permit a somewhat longer life to the thus afflicted individual, have caused scrutinising scientific attention to be given to the pathogenesis of these congenital heart lesions and their causal connection with the frequent co-existing developmental anomalies, as well as to their manifestations, intra vitam. One might remember the wonderful classical representation from John Hunter, in 1783, in which this keen observer, as the first after Morgagni, upon rational physiological basis, endeavored to explain the connection between congenital pulmonary stenosis and the malformation in the intraventricular septum, an almost constant accompanying phenomenon of this arterial constriction.

Hunter's investigations concerning the pulmonary stenosis tended to prove that the blood in fetal life, which, as a result of the obstruction in the course of the pulmonary artery, hindered in its passage through this normal canal, must necessarily have created an outlet from the right ventricle into the left, certainly then when the development of the septum between these ventricles was not completed. Therefore, according to Hunter's opinion, the constriction at the pulmonary orifice is the primary lesion, and the incomplete closure of the septum a result of the stasis of the blood in the right ventricle; in other words, Hunter established the well known mechanical theory of stasis.

Some years later Johann Meckel collected many of the already published cases of pulmonary stenosis, of congenital origin, published them in connection with a few of his own observations, refused to accept the stasis theory of Hunter as sufficiently explanatory and endeavored to demonstrate these developmental anomalies upon purely embryonal basis. Later the works of Dittrech, Peacock, and Heine helped to throw more light upon these previously little understood pathological conditions, as also did the brilliant exposition of H. Meyer, in Zurich, concerning the results of these congenital heart lesions upon the fetal circulation, particularly concerning the etiology of pulmonary stenosis. In his concluding remarks the last author says:

If we bring together the results of the whole examination, we are justified in advancing the sentence that in all cases in which incompleteness of the interventricular septum and narrowness, or obliteration, of the pulmonary artery occur near each other as defects in formation, the latter is always primary, and that when it is present, not only is the incompleteness of the intraventricular septum established but also the remaining coëxisting defects in formation in reference to the origin of the aorta, the foramen ovale, the ductus Botalli and the bronchial arteries. —Virchow's Archiv.

Kussmaul attempted in 1865 to assume a medium position and combine the embryonic and the stasis theory, to explain the causal connection between congenital pulmonary stenosis and the septum defect, and with this end in view published his well known "Correctur der Stauungs-Theorie."

In 1875, Rokitansky, as a result of his embryological investigations proposed an explanation entirely different, though by no means free from objections from those already taught for the origin of the septum defect, and endeavored to show that "the faulty division of the truncus art. communis, whose result is that a right position of the aorta occurs, is the primary arrest of development. Through this false position of the aorta the septum defect is caused, which in turn causes the faulty or insufficient conus formation."

If it be permitted a clinician to express himself concerning these questions of pure pathological-anatomical contents, I would conclude that from these theories that those from Kussmaul only could have general acceptation, particularly in the intermediary form; that at one time this method of occurrence, *i.e.*, the embryological developmental arrest, at another time, the process upon an inflammatory basis, primary pulmonary stenosis and secondary septum changes, would play a rôle. That the theory of stasis alone is not sufficiently explanatory appears from the following circumstances. We can picture to ourselves by a high degree of narrowing or complete atresia of the pulmonary artery only, that through the same, a change of pressure in the right ventricle occurs and as a result of which stasis and hindrance of the development of the septum become possible. By small or moderate degree of narrowing of the pulmonary artery such an explanation through stasis in the right ventricle and the purely mechanical working of the same upon the development of the septum cannot be accepted, since the right ventricle empties a small quantity only of blood into the pulmonary artery and through the ductus Botalli into the aorta. Against the solitary theory of embryological developmental arrest speaks the repeatedly advanced fact, that changes which prove to be of inflammatory nature in the fetal state were demonstrated in most cases thus far published.

Thus while the pathologists endeavored to explain the connection between these defects and the possible etiology of their origin, the clinicians did not keep equal pace with them. The literature of the cases of pulmonary stenosis of older date shows so great a disproportion between the clinical observations and their pathological-anatomical investigations that it speaks for a great deficiency in corresponding clinical interest, and caused Freymann to remark, "that accurate clinically observed pure congenital heart lesions belong to the rarities." It is thus not surprising when one considers that the observations were made principally upon children without an effort anatomically to accurately localise the lesion, and the generally advanced or accepted diagnosis of a congenital heart lesion sufficed.

In many cases of pure pulmonary stenosis the clinical diagnosis could be made without difficulty, because the classical signs allowed hardly another interpretation, but the more accurate diagnosis of the developmental anomalies which accompany them has become, on account of deficiency in explanation of the physical signs present, a subject of great speculation and a source of frequent error. For instance, how many cases do we find reported of pulmonary stenosis, in which on the theoretical basis, that because the second pulmonary tone was found accentuated the probable diagnosis of a persistent and open ductus Botalli was made and in which the autopsy failed to reveal even a trace of a persistent ductus Botalli, but demonstrated an open foramen ovale?

It is in reference to this lack of harmony between the clinical manifestations of these cases and the autopsy results and the heretofore universally taught pathognomonic signs that we feel ourselves not only justified but forced from the clinical observations of three classical cases of congenital pulmonary stenosis to report them in detail. It will show better how in each of them the socalled pathognomonic clinical signs though eminently present were found by the autopsy to have been utterly misleading and extremely confusing, particularly the constant presence of a remarkable accentuation of the second pulmonary tone, which caused the intra vitam diagnosis of persistent and open ductus Botalli to be made, while post mortem no trace of a persistence of this fetal communication could be demonstrated.

I will append herewith the detailed clinical histories, together with the post-mortem findings of these cases as additions to the literature of this subject, and upon the basis of their constant clinical phenomena and an analysis of the accessible previously published cases of this nature, attempt to portray a complete typical, sharpely defined clinical picture of congenital pulmonary stenosis in the adult and to discuss in detail the diagnostic possibilities of the co-existing developmental anomalies. I would like to remark here that I found myself necessitated to deviate from the generally accepted theory that the predisposition to that so frequent, complication in these heart lesions, the pulmonary tuberculosis, is a result of a stenosis in the pulmonary artery, and instead of this to propose a better established and more acceptable theory—namely, that this predisposition is due to an anemia of the lung tissue, a result of a co-existing congenital narrowing of the aortic system.

Case I.—S.M., female, aged 17; accepted July 30, 1896; died October 3, 1896.

*Anamnesis.*—The father and one brother of the patient died of pneumonia; the mother of heart disease. Two brothers and one sister are living and enjoying good health. The patient affirms that she always up to her tenth year enjoyed good health, although her lips were always bluish and the color of the skin was always pale. At the age of ten she remarked for the first time that upon the slightest exertion she would become short of breath. This condition lasted until about two years ago, at that time heart palpitation and cough began to manifest themselves. The expectoration was profuse and greenish-yellow. Although she was always anemic and poorly developed she maintains that she has become emaciated and weaker during the past two years. At the present time she complains of shortness of breath, heart palpitation and harrassing cough. These increase upon the slightest movement. The expectoration is very copious, of a greenish-yellow color. The appetite is poor, bowels very constipated. The patient has never menstruated, and has never had rheumatism or any other infectious disease.

*Status Presens.*—The patient is small, gracile, poorly developed, very much emaciated, of slight muscular development and fat polster. She is very anemic. The both breasts are very small, undeveloped, crenes pubis sparsely present. The pulse is small, equal, rhythmic. Temperature, 102½° F.; pulse, 96; respiration, 32.

*Head.*—The head is small, well formed, moves freely. Nothing abnormal concerning the nose and ears. The hair is not profuse. The visible mucous membranes are pale, at the same time there exists a trace of cyanosis. In the mouth nothing abnormal. The cheeks present a circumscribed redness; the both pupils are equally wide, react promptly to light and accommodation. The movements of the eyes free.

*Neck*.—The neck is long, lean, shows no pulsation, no swelling of the glands.

*Thorax*.—The supra- and infraclavicular grooves upon both sides sunken in. The left side does not expand; there are no retractions of the intercostal spaces. Percussion: over both apices dull percussion note, particularly posteriorly. On the right side, anteriorly the dulness extends to the upper border of the fourth rib, posteriorly to the middle of the scapula; from this point downward, resonant note to one handbreadth under the lower angle of the scapula, the lung border is mobile by respiration. On the left side anteriorly, the short percussion note extends to the third rib, posteriorly to the middle of the scapula; from here downward, clear note to the normal limit, mobile by respiration. The vocal fremitus over the areas of dulness is increased, over the remaining parts distinctly present.

Auscultation: Over both apices, on the right to the fourth rib anteriorly, on the left to the third rib, posteriorly to the middle of the scapula, rough bronchial inspiration, metallic râles, prolonged bronchial expiration. Over the remaining portions puerile, vesicular breathing.

*Heart*.—The apex-beat lies in the fifth intercostal space, in the mammary line, is diffuse, undulating. In the second intercostal space, left from the sternum, one can feel a rough systolic purring—fremissment—which can be traced into the left shoulder. At the apex, a slight fremissment and undulation. The heart dulness begins above on the upper border of the third rib, to the left at the apex-beat, extends to the right to one fingerbreadth over left sternal border. Auscultation: at the apex one can hear a soft blowing systolic murmur, which is transmitted into the axilla, and posteriorly to the angle of the scapula; the second tone is clear. From here upward toward the base the murmur increases in intensity until over the pulmonalis, second intercostal space to the left, where one hears a pre- and systolic rough sawing murmur, which is distinctly transmitted into the left shoulder, but is not heard in the carotids; the second pulmonary tone is distinctly accentuated; as if hanging on to the second tone is a distinct diastolic murmur. The second aortic tone to be heard.

*Abdomen*.—The liver is not painful, not enlarged. The spleen not accessible. No edema of the lower extremities.

*Sputum*.—Tubercle bacilli richly demonstrable.

*Urine*.—Normal.

*Decursus Morbi*.—August 8, the lower extremities beginning to swell; the liver somewhat enlarged, painful; the cough still embarrassing; expectoration very copious. August 17, on the left side, just above heart, distinct physical phenomena of cavity demonstrable. October 1, the liver reaches to umbilicus, very sensitive upon pressure; edema of lower extremities increasing. October 3, death occurs under the picture of extremest orthopnea.

*Post-mortem Findings.*—October 3, external appearances correspond to the clinical description. In both lung apices very large cavities of tuberculous nature. Venous hyperemia of all the other internal organs.

*Heart*—(Dr. Landsteiner, of Vienna,) Length of the heart from the apex to the aortic orifice, 8 c.m. The size of the heart upon the whole corresponds somewhat to the size of a man's fist. The left ventricle roomy, the thickness of its wall, 0.9 c.m. The right ventricle a little smaller than the left, greatly hypertrophied, the thickness of its wall, 1 c.m. The walls of the right auricle thicker than those of the left. The aortic, mitral and tricuspid valves normally developed. Circumference of the aorta just above the valves measures 5.5 c.m. The circumference of the pulmonary artery, 2 c.m., above the valves, 7.5 c.m. The three cusps of the pulmonary valves are grown together in a regular manner, their borders are thickened and rounded, the opening is considerably constricted by these adhesions. By this growing together, a sort of a diaphragm, with a circular opening, in the middle is formed this opening has a diameter of 1.2—1.3 c.m. Between the valves and the wall of the pulmonary artery, deep sinuses. The conus of the pulmonary artery has a circumference of 4.5 c.m. The septum between the ventricles complete, no defect demonstrable. In the auricular septum, the foramen ovale is found wide open; the valvula foramen ovale is developed. Behind the foramen ovale, above the entrance of the ven. Thebesii, is a defect 9 m.m. long by 4 m.m. wide. This defect is oval shaped and has its greatest axis situated about parallel with the ring of the tricuspid valves.

*Diagnosis*—Stenosis and insufficiency of the pulmonary valves, open foramen ovale, defect in auricular septum, hypertrophy of the right ventricle of heart, Angusta aorta.

*Epicrisis of Case I.*—In this case we had to do with a gracile, weakly developed individual, who had never had an infectious disease; since she was somewhat stunted in her development—no menstruation, failure of crenes pubis, lack of development of breasts—and since her childhood had been cyanotic, the possibility was at hand that we had to do with a congenital heart lesion.

The constant physical findings: (*a*) remarkably strong systolic fremissment over the whole heart, most distinct over the second intercostal space to the left of the sternum, which could be followed into the left shoulder; (*b*) distinctly demonstrable enlargement of the heart to the right; (*c*) systolic sawing, rough murmur, most intense in the second intercostal space, left from the sternum, which was transmitted into the left shoulder, accompanied by a diastolic murmur, which closed itself upon a remarkably loud, accentuated diastolic tone; (*d*)

the proof of extensial tubercular infiltration of both lung apices;—justified our clinical diagnosis of stenosis and insufficiency of the pulmonary valves, congenital in nature, and on account of the accentuation of the second pulmonary tone a persistence and patulous ductus Botalli. The autopsy revealed no sign of a persistence or remaining open of the ductus Botalli, but a wide open foramen ovale and defect in auricular septum.

Case II.—K. L., book-binder; single; 22 years of age; accepted October 22, 1899; died October 25, 1899.

*Anamnesis*—The patient's father died from some disease of the chest, at the age of 46 years; his mother from pneumonia at the age of 42 years; two brothers died in childhood, one from diphtheria; cause of other's death unknown. Patient himself had during childhood scarlet fever, smallpox, typhoid fever, and three times pneumonia; in 1893 pleuritis. Cough has existed since childhood, dyspnea and palpitation as long as patient can remember. Patient affirms that he has always been as weak as at present; he has never had rheumatism. Alcohol very little; syphilis denied. Since the past four months increase of complaints, greater cough and more copious expectoration, more evident heart palpitation and intense dyspnea.

*Status presens.*—The patient is less than medium size, greatly emaciated, strongly cyanotic, particularly in peripheral parts; the visible mucous membranes very anemic; the eyes somewhat prominent, the pupils alike moderately wide, react somewhat slowly to light and accommodation; movements of eyes free. The teeth very defective, very much deformed. Skull somewhat hydrocephalic and rhachitic. The arteries soft, straight, the tension normal, the pulse wave low, the frequency 96, equal and rhythmical. The neck small and long, slight struma, distinct undulations of veins of neck visible, negative venous pulse.

*Thorax.*—The thorax is very long, small, very flat; the inspiration reaches its height after many interruptions. On the left side the breathing over the whole lung is bronchial, in places very weak, with loud metallic râles, distinct friction rub. All over, one can hear the heart tones, particularly over the left apex; the second tone appears as a murmur.

*Heart.*—The whole cardiac region pulsates distinctly; the apex-beat is in the fourth intercostal space, within the mammary line, distinctly visible. From this point toward the base and downward an undulation is transmitted, which ceases at second intercostal space to the left of the sternum.

The apex-beat can be felt distinctly, slightly heaving; can be covered by one finger tip. At the apex one feels a very short systolic and a diastolic fremissment. This fremissment is transmitted upward in the neighborhood of the clavicula and is most distinct over pulmonalis. The cardiac dulness begins on the left at the mammary line, extends to the right a little over the left sternal border.

At the apex one hears a very rough, loud systolic murmur, the second tone very loud, clear. Over the pulmonalis one hears a very short pre- and systolic murmur, a very loud, accentuated second pulmonary tone, and an uncommonly long, hissing diastolic murmur. Over the aorta, two clear tones.

*Lungs.*—Intercostal spaces, supra- and infraclavicular grooves very much retracted. The left mamma higher than the right, precardial region more prominent than the rest of the thorax. The respiration is costo-abdominal; distinct movements of alænasi. The percussion note over both apices strongly dull, on the left more so than on the right; distinct tympany. The right lung reaches the upper border of the seventh rib, the mobility lessened. On the left side, in the first and second intercostal spaces, the note is dull, tympanitic; the lung reaches as far as upper border of the fourth rib, yet the heart dulness is difficult to differentiate from the dulness of the lung. On the right side the breathing is bronchial, moist, large and small râles in inspiration and expiration. Laterally pleuritic friction murmur can be heard on the left side, amphoric breathing, large metallic clinging râles.

Posteriorly over both apices, note dull, tympanitic, the left more tympanitic than the right. The right lung extends downward to one hand breadth under the angle of the scapula, very slightly mobile. On the left side the tympanitic note extends to the angle of scapula, from here downward dull, retains tympanitic character. On the right over the apex, sharp vesicular breathing; downward the breathing becomes bronchial, with loudly metallic sounding râles.

*Abdomen*, on level with thorax. The liver dulness greatly enlarged. The liver palpable. The spleen is enlarged. There is no ascites, no edema.

Beginning clubbing of fingerends.

*Autopsy Diagnosis.*—Excentric hypertrophy of the right ventricle and right auricle, with open foramen ovale. Hypoplasia of the aorta and peripheral arteries. Mechanical hyperemia of internal organs. Chronic tuberculosis of lungs, with cavities; adhesive pleurisy, left side. Tubercular ulceration of small bowels. Stenosis and insufficiency of pulmonary valves.

*Epicrisis of Case II.*—In this case the same symptom-complex as characterised case I. justified the diagnosis of a congenital valvular stenosis and insufficiency, and on account of the remarkably loud accentuated second pulmonary tone the diagnosis of a persistent and open ductus Botalli. In this case not a remnant of a ductus Botalli, but a wide open foramen ovale, was present at the autopsy.

Case III.--J. B., 21 years of age; accepted January 31, 1901; died March 5, 1901.

*Anamnesis.*—The father died after a fourteen days illness, of pneumonia; mother lives and enjoys good health. Patient has four sisters, all of whom are well.

The patient remembers that since his earliest childhood he suffered from dyspnea upon the slightest exertion, particularly when running or ascending stairs, and that his lips were always blue. He never noticed heart palpitation.

Four years ago he had pneumonia and pleurisy. During that attack he was confined to bed two months. Since this time he has noticed heart palpitation and increasing dyspnea upon the slightest muscular exertion. In March last year he expectorated blood. In November and December had severe cough with yellowish green expectoration. Since then the dyspnea has greatly increased and the palpitation become more perceptible.

At present the patient complains of shortness of breath and severe heart palpitation, night sweats and general weakness. Lately he has become markedly emaciated. He has never had rheumatism, nor any other infectious disease.

*Status presens.*—The patient is greatly emaciated, the skin-color, anemic. He possesses a rather good bony development, slight musculature and little fatty tissue. Temperature, p.m., 103° F., a.m., 36.6° F. Pulse 114, dicrotic, easily compressible, the artery small.

*Head.*—The skull is well formed, shows no abnormalities. Both pupils are normal, equal upon both sides, react promptly to light and accommodation. The movements of the eyes are free. The lips are anemic, cyanotic, livid; mouth, teeth and throat normal. Upon both cheeks is a circumscribed redness; mucous membrane of throat very pale. Tongue is moist, slightly coated. There is no difficulty in swallowing.

*Neck.*—The neck is long, small. No visible pulsation. Glands enlarged, not infiltrated. Both carotids pulsate alike, no purring to be felt. Arteries small; no distinct pulsation in the jugulum.

*Thorax.*—The breathing is costa-abdominal, dyspneic; the thorax is long, small, flat. Supra- and infraclavicular retractions on both sides. The fifth and sixth intercostal spaces on the right, retracted in inspiration. On the left side the eighth and ninth intercostal spaces retracted. The right side participates more in the respiration than the left. Tendency to "funnel-breast." Percussion: Anteriorly on the right note is dull tympanitic to third rib. From this point downward to the upper border of the sixth rib, good mobility—in para sternal line. In the axillary line as far as the sixth rib, good percussion note, then slight dulness, respiratory mobility not present. Posteriorly, over both apices dull, tympanitic note, which reaches the middle of scapula. From here clear note to one hand's breadth below angle of scapula. Mobility not present. On the left side, dull, tympanitic note as far as upper border of second rib. In the axillary line, resonant note as far down as the ninth rib. Posteriorly over apex, dull tympanitic note. Distinct change of note upon opening and closing of mouth. The dulness extends to the middle of the scapula, from here downward, clear note to three finger breadths below the angle of the scapula; not mobile. Beginning at the lower border of the second rib, intimately along the left sternal line is a 2 c.m. wide dulness which extends downward and merges into the heart dulness. This dulness forms a triangle with the apex above on the lower border of the second rib.

Auscultation of lungs: In the right anteriorly to the third rib, bronchial inspiration and expiration, râles moist and metallic. Downward the bronchial breathing lessens in intensity. On the left, amphoric breathing as far as the second rib, metallic râles. In the axilla bronchial breathing. Posteriorly on the right, bronchial breathing, metallic râles, downward less intensial. Left, amphoric metallic, breathing metallic râles. The heart tones sound very metallic on this part. Over the rest of the lungs, rales and bronchial breathing to be heard.

*Heart.*—In the left lateral position the apex beat lies in the fifth intercostal space, two finger breadths outside of mammary line, not heaving. In the second intercostal space to the left of the sternum, one can feel a rough, very intense systolic fremissment, and a clapping second pulmonary tone. This fremissment is transmitted in the second interspace into the left shoulder, but not into the carotids. Percussion: Absolute dulness begins above on the upper border of the third rib, on the left at position of the apex, on the right as far as one finger breadth over left sternal border. The lung borders are promptly mobile by respiration. Auscultation: at the apex, dull systolic murmur; second tone clear, not accentuated. In the middle of the heart's dulness the systolic murmur assumes a rougher character; second tone loud, almost accentuated. At the junction of the third rib and sternum, murmur and tone more intense. In the second intercostal space to the left of the sternum, over pulmonalis one hears most distinctly an intensely loud rolling, systolic murmur, and remarkably accentuated clear pulmonary second sound; both murmur and tone are transmitted into left shoulder, and not so loud in the carotids. Over the aorta one hears the first sound, systolic murmur; second sound well rounded. Over the tricuspid the same finding. The systolic murmur can be heard all over left thorax.

*Liver.*—Not enlarged, not painful.

*Spleen.*—Cannot be felt, not enlarged, no edema.

*Blood.*—Shows secondary anemia, otherwise nothing remarkable.

*Urine.*—Normal with exception of presence of urobilin and skatol.

The "decursus morbi" of this case is so long and as it adds no light to the diagnosis, I shall omit it. I will, add, however, that repeatedly a distinct pre-systolic murmur could be heard over pulmonalis.

The patient died on March 5, 1901, under the picture of the most intense cyanosis and orthopnea.

*Post-mortem.*—Extensive tuberculous cavities in both lungs; adhesions of both pleuræ. Pulmonary stenosis and insufficiency, conus and valves stenosed. Foramen ovale persistent and open. Defect in interventricular septum. Hypertrophy of right ventricle, right and left auricles. Dilatation of auricles, particularly the right. Dilatation of pulmonary artery above the valves; congenital narrowness of the aorta. Passive hyperemia of the internal organs.

*Epicrisis to Case III.*—The clinical phenomena which this case presented deviated very little from the preceding two; on account of the remarkable accentuation of the second pulmonary sound the presence of a persistent and patulous ductus Botalli with conus stenosis of pulmonalis, was diagnosticated; on account of the age (21 years) of the patient, the uncommon loudness of the first tone over the pulmonary orifice and the systolic murmur in the vessels of the neck, a septum defect was presumed. At the autopsy there was not a sign of a persistent ductus Botalli; instead of this was a foramen ovale open, and a defect in the intraventricular septum.

Here were three cases of congenital pulmonary stenosis which presented themselves clinically in an eminently classical manner, in all of which an account of the permanent presence of a remarkable accentuation of the second pulmonary sound, despite the fact that a conus and valvular stenosis existed, upon the authority of the theoretical deductions of the physiologist the ductus Botalli was diagnosticated as persistent and patulous, and in all three cases not even a trace of this fetal communication was demonstrable at the autopsy. Upon the other hand, the foramen ovale was open in every one of them.

In order to facilitate our task it would be more to the purpose to accept the stasis theory as the most plausible, the pulmonary stenosis as the primary lesion and to study what purely mechanical phenomena would result in the fetal circulation, and then in the extra-fetal, in a case of congenital pulmonary stenosis of moderate degree.

Concerning the fetal conditions normally it will suffice to follow the blood from the inferior vena cava to its final destination in the aortic system. From the inferior vena cava it passes into the right auricle, where, guided by the Eustachian valve it is directed into the left auricle through the foramen ovale. In the fetus the Eustachian valve is well developed and disappears after birth, its disappearance beginning with the suspension of its function, the closure of the foramen ovale. All of the blood which comes into the right auricle does not pass through the foramen ovale, but a small part passes through the auriculo-ventricular opening into the right ventricle from which it is pumped into the pulmonary artery by the ventricular systole; here instead of going into the lungs it passes through the ductus Botalli into the aorta. Thus the two streams of blood formed at the Eustachian valve meet again and form one column of blood.

Now let us suppose, for instance, that the small amount of blood which passes through the pulmonalis would be hindered, to a certain degree, by a stenosis of the opening of this artery. What results would such a condition cause? We proceed from the consideration that a case of moderate degree would show these results more distinctly than a case of atresia, and therefore will leave the latter out of the question. In the adult those cases only interest us which are of a moderate degree of stenosis; the cases of atresia we will therefore not bring into discussion, particularly above all on account of the brevity of observation which these cases present—they die mostly within a few weeks. It will be clear that these results depend upon the time of the origin or existence of the constriction and the extent of the anatomical changes. As the first result we find a stasis in the right ventricle. The result of this is that the venous blood would flow through the foramen ovale in greater quantities into the left auricle, since it passes in the direction of the lower pressure. Further, the stasis in the right ventricle would bring about a hypertrophy, which through its increased power can allow at least a quantity of blood equal to the normal, to pass through the stenosis. In concension, it can give rise to a "Dehiscenz" in the interventricular septum if it occur before the complete development of the septum. We thus see that from a purely physical consideration the pulmonary stenosis must have, under all circumstances, a certain influence upon the development of a septum defect or of an open foramen ovale. If hypertrophy of the right ventricle with increased working power occur, a result of the presence of a stenosis of the pulmonalis, therefore can have no influence upon the remaining open of the ductus Botalli, a lesser degree of development of a hypertrophy would have a preventative influence.

An effect upon the septum defect would depend upon the time in which the stenosis of the pulmonalis occurs. Therefore in a case of congenital pulmonary stenosis in order that complete compensation in the fetal circulation take place, either a corresponding hypertrophy of the right ventricle must occur, or if this does not happen, the blood must find another outlet than through the pulmonary artery and the ductus Botalli, either by an increased flow through the foramen ovale into the left auricle, or through a defect in the interventricular septum or through both, and hence the ductus Botalli as a canal of communication from the right ventricle into the aorta would have very little function; in fact probably less than normal. One can very easily see that from a purely mechanical standpoint in pulmonary stenosis the remaining open of the ductus Botalli is a seldom occurrence, an assertion for the support of which we can quote statistics, which say that the ductus Botalli appears open correspondingly seldom in pulmonary stenosis. On the contrary, the foramen ovale appears very frequently, a fact which agrees with our resumé.

After we have considered the results of such a constriction of the pulmonary artery in the fetus, we can turn our attention to the results which take place in the circulation when the constriction at the ostium pulmonale persists. If the stenosis be complete, in order that the circulation remain in a state of compensation there must occur one of two things, either (1) the ductus Botalli must necessarily remain open, and either the foramen ovale or defect in the septum or both, must remain in function; the right ventricle can remain either hypertrophied or atrophied; (2) the individual must immediately die. In the cases where the stenosis is not complete—and these are the ones which interest us —the compensation depends on the hypertrophy of the right ventricle or on the existence of these defects, —namely, on the remaining open of the foramen ovale or on defect in the interventricular septum, or on both.

In the cases where the stenosis of the pulmonalis is patulous and a compensatory hypertrophy of the right ventricle exists, the ductus Botalli need not have any further function, and could persist as a very seldom occurrence after birth, a well known fact to which the previously published cases attest. There are, therefore two possibilities for the compensation of a congenital pulmonary stenosis of moderate degree to consider: (1) Corresponding hypertrophy of the right ventricle without persistence of the fetal canals; (2) through congenital defects; the second of which is present in the great majority of cases.

We have considered in the foregoing the pure results of the pulmonary stenosis and the influence which it has upon the circulation. If we use these purely mechanical phenomena as a bridge to the symptoms, we arrive firstly at such which depend on the situation of the stenosis, then, as is well known, there are infra-valvular, valvular and supra-valvular stenosis. We will later have occasion to mention in our discussion of the clinical symptoms, that according to the localisation of the stenosis, one or the other symptom changes.

[*Concluded in September number.*]

